# Disruption of *Yarrowia lipolytica* biofilms by rhamnolipid biosurfactant

**DOI:** 10.1186/2046-9063-8-17

**Published:** 2012-07-27

**Authors:** Devendra H Dusane, Sushovan Dam, Yarlagadda V Nancharaiah, Ameeta Ravi Kumar, Vayalam P Venugopalan, Smita S Zinjarde

**Affiliations:** 1 Institute of Bioinformatics and Biotechnology, University of Pune, Pune, 411 007, India; 2 Biofouling and Biofilm Processes Section, BARC Facilities, Kalpakkam, 603 102, India; 3 Present address: Biocolloids and Surfaces Laboratory, Department of Chemical engineering, McGill University, Montreal, QC, Canada

**Keywords:** Biofilm, Biosurfactant, CTAB, Rhamnolipid, SDS, *Yarrowia lipolytica*

## Abstract

**Background:**

*Yarrowia lipolytica* is an ascomycetous dimorphic fungus that exhibits biofilm mode of growth. Earlier work has shown that biosurfactants such as rhamnolipids are efficient dispersants of bacterial biofilms. However, their effectiveness against fungal biofilms (particularly *Y. lipolytica*) has not been investigated. The aim of this study was to determine the effect of rhamnolipid on a biofilm forming strain of *Y. lipolytica*. Two chemical surfactants, cetyl-trimethyl ammonium bromide (CTAB) and sodium dodecyl sulphate (SDS) were used as controls for comparison.

**Results:**

The methylene blue dye exclusion assay indicated an increase in fungal cell permeability after rhamnolipid treatment. Microtiter plate assay showed that the surfactant coating decreased *Y. lipolytica* biofilm formation by 50%. Rhamnolipid treatment disrupted pre-formed biofilms in a more effective manner than the other two surfactants. Confocal laser scanning microscopic studies showed that biofilm formation onto glass surfaces was decreased by 67% after sub-minimum inhibitory concentration (sub-MIC) treatment with rhamnolipids. The disruption of biofilms after rhamnolipid treatment was significant (P<0.05) when compared to SDS and CTAB.

**Conclusion:**

The results indicate a potential application of the biological surfactant to disrupt *Y. lipolytica* biofilms.

## Background

*Yarrowia lipolytica* earlier referred to as *Endomycopsis lipolyticaSaccharomycopsis lipolytica* or *Candida lipolytica* is a hemiascomycetous fungus belonging to the Saccharomycetales order. It is often isolated from environments that are rich in hydrophobic substrates [1]. The organism inhabits soil [2], seawater [3] and refrigerated meat products [4]. The fungus is found in the oral cavity, pulmonary tract and intestines of healthy individuals. It is also an opportunistic pathogen that causes oral candidiasis, candidemia and catheter related infections [5]. From biomedical point of view, the eradication of this organism thus becomes important. The fungus forms biofilms on different surfaces in the presence of a variety of substrates [6]. It is well known that microorganisms in the biofilm mode of growth often resist a variety of antimicrobial agents. There is thus a need to explore alternative means of disrupting biofilms. A variety of chemicals including biocides and surfactants have been used to control biofilms [7]. Chemical surfactants find applications in areas of medical care. For example, cetyl trimethyl ammonium bromide (CTAB) is used as a disinfectant in medical settings [8]. Sodium dodecyl sulfate (SDS) is effective by mediating leakage of cellular material from microorganisms [9].

Widespread use of chemical surfactants is discouraged due to their inherent toxicity. In this context, biosurfactants are being favored [10]. The latter group of surfactants offer several advantages in being relatively non-toxic, effective under different environmental conditions and in being biocompatible [11,12]. Biosurfactants have been used to disrupt bacterial biofilms [13,14]. However the reports on the efficacy of biosurfactants on fungal biofilms are limited [15]. We hypothesized that rhamnolipids may be effective against biofilms of *Y. lipolytica*. The yeast strain used in the current investigation forms biofilms on a variety of water-soluble and -insoluble substrates. The objective of this work was therefore to test the effectiveness of rhamnolipids in (i) preventing biofilm formation and (ii) in disrupting pre-established biofilms of *Y. lipolytica*. The results have been compared with two chemical surfactants.

## Results and discussion

### Minimum inhibitory concentration (MIC) and minimum fungicidal concentration (MFC) values of surfactants

The chemical and biological surfactants displayed antifungal activity against the cells of *Y. lipolytica* NCIM 3589. Rhamnolipid and CTAB displayed a minimum inhibitory concentration (MIC) of 5% ± 0.1 w/v and minimum fungicidal concentration (MFC) value >10% ± 0.1 w/v, while SDS showed MIC and MFC values of 0.62% ± 0.05 w/v. SDS was more effective as an antifungal agent compared to rhamnolipids and CTAB. This anionic surfactant is known to possess detergent and antimicrobial properties [16]. The surfactant permeabilizes cells by targeting the cytoplasmic membranes and by affecting membrane-bound enzymes [16]. Rhamnolipids are anionic biosurfactants that disrupt cells by interacting with the phospholipid components of the biological membranes [17,18]. Rhamnolipids derived from *Pseudomonas aeruginosa* are known to possess antifungal activity against some plant pathogenic fungi such as *Cercospora kikuchii, Cladosporium cucumerinum, Colletotrichum orbiculare, Cylindrocarpon destructans, Magnaporthe grisea* and *Phytophthora capsici*[19]. The rhamnolipids inhibited spore germination and prevented hyphal growth in *P. capsici* at concentrations of 50 μg ml^−1^. A cationic surfactant, CTAB displayed lower antifungal activity towards *Y. lipolytica* as compared to SDS or rhamnolipids. In the present investigation, lower antifungal activity of CTAB could be a result of reversal of fungal cell surface charge and not due to cell lysis, as observed with SDS [9].

### Increase in cell permeability after treatment with surfactants

**Figure 1:**
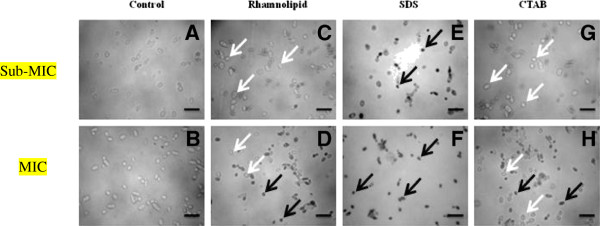
**Morphological features of *Y. lipolytica* NCIM 3589 cells.** Control cells (**A** and **B**); after treatment with rhamnolipid (**C**, **D**); SDS (**E**, **F**); and CTAB (**G**, **H**) at sub-MIC and MIC concentrations, respectively, for 1 h. The sub-MIC and MIC concentrations of 2.5% and 5% w/v respectively for rhamnolipid and CTAB and 0.3% and 0.62% w/v concentration of SDS were used. Bar indicates 10 μm. The upper panel indicates sub-MIC concentrations and lower panel shows MIC concentrations of the surfactants tested. White arrow shows non-permeability of methylene blue within the yeast cells whereas the black arrow depicts increase in cell permeability and uptake of the dye after treatment with surfactants.

Rhamnolipids display antimicrobial and surfactant properties [14]. They are known to increase cell permeability of *P. aeruginosaEscherichia coli* and *Bacillus subtilis*[20]. In the present case, rhamnolipids were found to be less effective in increasing the cell permeability of *Y. lipolytica*, as compared to SDS. SDS displayed higher permeability even at concentrations lower than MIC values (Figure 1, Table 1). In Figure 1, white arrows point towards non-permeabilized cells and the black arrows depict permeabilized cells. The increase in permeability observed after treatment with SDS may be due to the possible formation of molecular aggregates in the membrane and creation of trans-membrane pores [21]. CTAB was least effective in permeabilizing cells. This reduced permeability of the dye could be due to the robust nature of the fungal cell walls [22].

**Table 1:** **Methylene blue dye exclusion by *Y. lipolytica* cells post treatment with ½ MIC and MIC of surfactants for 1 h**

**Surfactants**	**Cells stained at ½ MIC (%)**	**Cells stained at MIC (%)**
Rhamnolipid	20 ± 2	25 ± 2
SDS	70 ± 4	95 ± 2
CTAB	7 ± 2	14 ± 4

### Effect of surfactant pre-coating on biofilm growth

**Figure 2:**
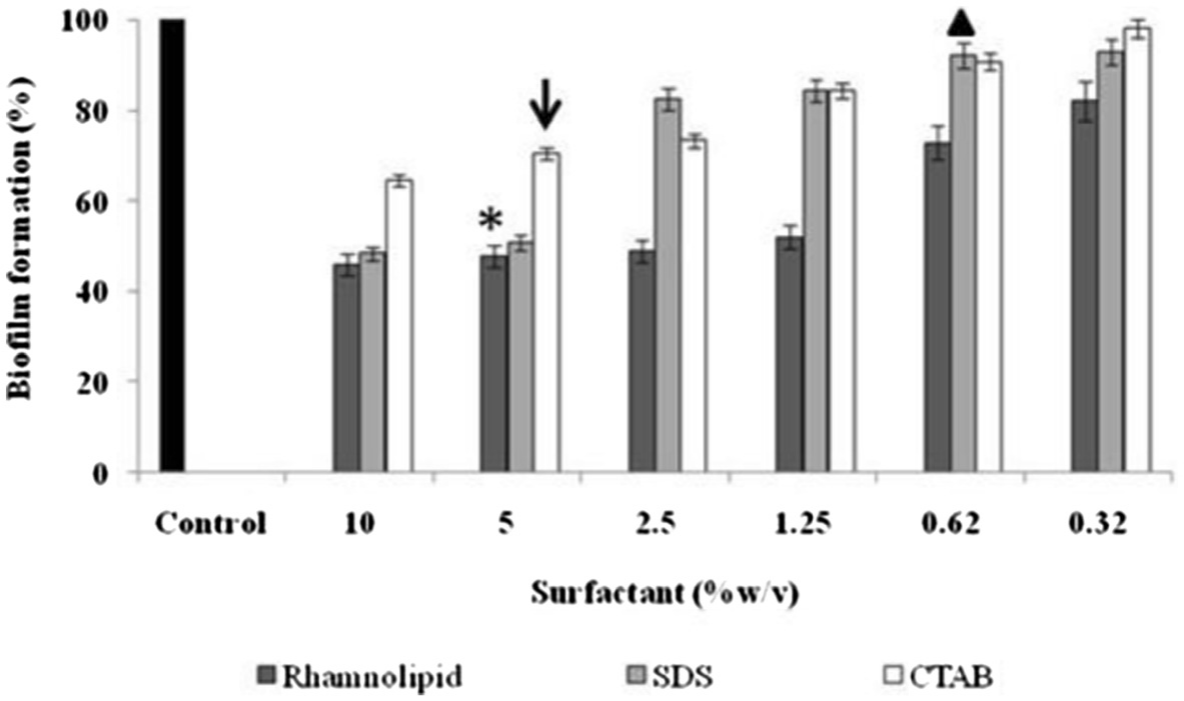
**Inhibition of *Y. lipolytica* NCIM 3589 biofilms by surfactants.** Observations in microtiter plate wells after pre-coating with different concentrations of rhamnolipid, SDS or CTAB. Control indicates biofilm formation by *Y. lipolytica* in microtiter plates untreated with the surfactants. The OD values are normalized with reference to control (considered as 100%). Where asterisk (*), triangle (▴) and arrow (↓) indicates MIC concentrations of rhamnolipid, SDS and CTAB respectively.

Anti-adhesive activity of microbial surfactants has been reported earlier [11,14,23]. Pre-coating of microtiter plate wells with the surfactants effectively reduced the development of *Y. lipolytica* biofilms. Adhesion of *Y. lipolytica* cells to the microtiter plate wells was inhibited to 50% with rhamnolipids at MIC concentration (5%) (Figure 2, asterisk). Rhamnolipids showed significant anti-adhesive ability (P<0.05) as compared to CTAB that inhibited 29% at MIC value of 5% (Figure 2, black arrow). With SDS at MIC (0.625%) the inhibition was less than 10% (Figure 2, black triangle). Rhamnolipids are known to decrease *Listeria monocytogenes* attachment when preconditioned onto Polytetrafluoroethylene (PTFE) surfaces [24]. We have also recently demonstrated the ability of rhamnolipids in disrupting *Bacillus pumilus* biofilms by removing exopolymeric substances [14]. SDS is known to display anti-adhesive ability by affecting the hydrophobic bonds that help in attachment of cells to the surfaces [25]. In the present study however, SDS showed lower anti-adhesive ability than rhamnolipids. Although CTAB is reported to bind to the negatively charged microbial surfaces, alter their surface charge and prevent the binding of the cells to the surfaces [26], this surfactant was not as effective in preventing *Y. lipolytica* adhesion. In the present study, the anti-adhesive effect of rhamnolipids was found to be significantly higher as compared to SDS and CTAB suggesting the potential of rhamnolipids as anti-adhesive agents in the treatment of fungal biofilms.

### Disruption of preformed biofilms of *Y. lipolytica*

**Figure 3:**
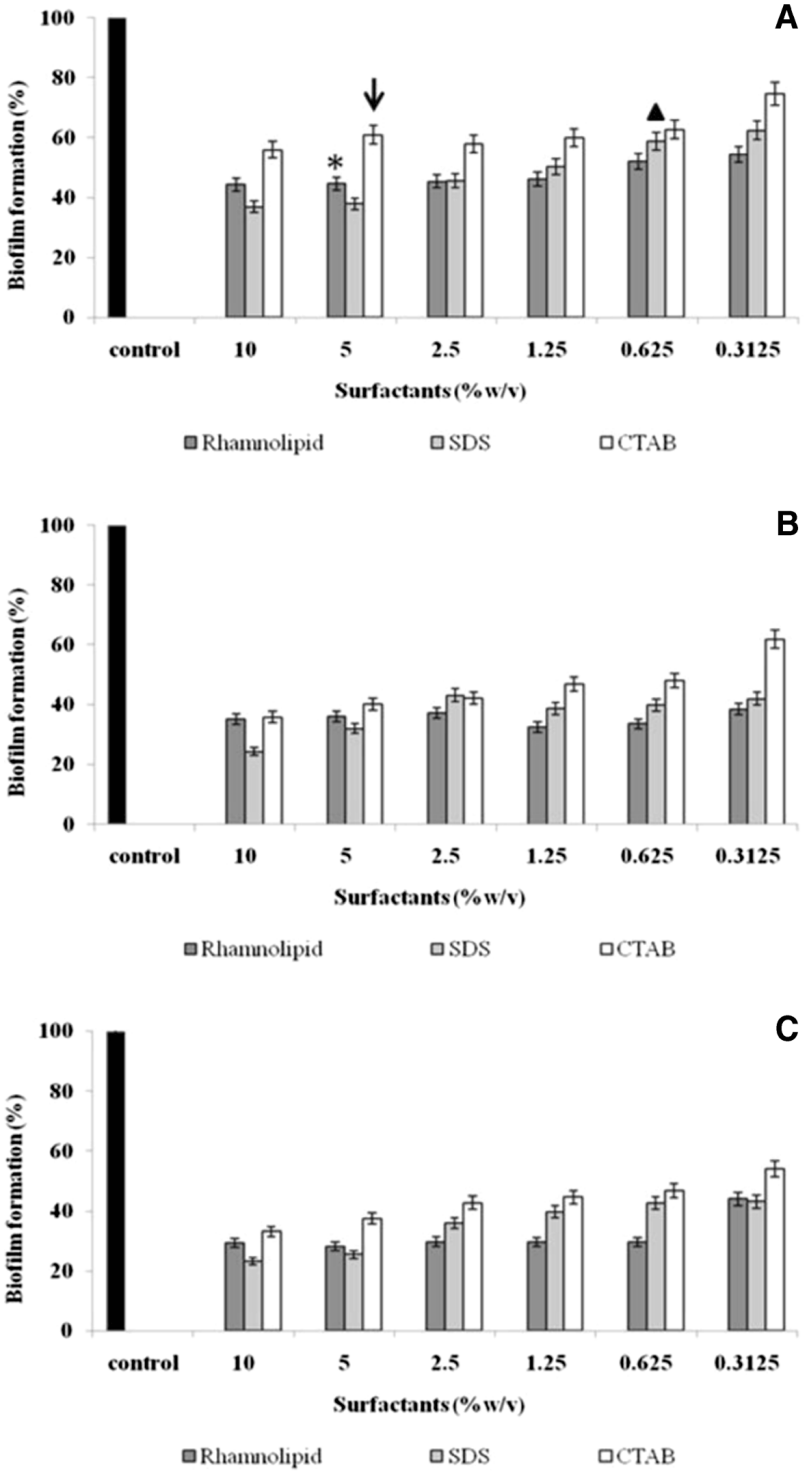
**Effect of surfactants on preformed biofilms of *Y. lipolytica* NCIM 3589.** Observations with rhamnolipid, SDS and CTAB after (**A**) 1 h (**B**) 2 h and (**C**) 3 h of incubation. Where, star (*), triangle (▴) and arrow (↓) indicates MIC concentrations of rhamnolipid, SDS and CTAB respectively.

The preformed biofilms of *Y. lipolytica* were treated with the surfactants for 1, 2 or 3 h. Biofilms of *Y. lipolytica* formed for 3 days in microtiter plate wells were disrupted effectively (55% with rhamnolipid, 35% with CTAB and 40% with SDS at respective MIC values) within 1 h of treatment with the surfactants (Figure 3a asterisk, arrow and triangle, respectively). At concentrations of surfactants lower than MIC, rhamnolipid displayed effective dispersion of biofilms (46%), followed by SDS (38%) and CTAB (25%). At higher concentrations (>2.5%), the effect of SDS was slightly better than that of the rhamnolipid. However, over a period of time, the efficacy of rhamnolipids was found to be similar to that of SDS (Figure 3b and 3c). CTAB was less effective in controlling biofilms, possibly due to its chemical interaction with fungal proteins present in the exopolymeric matrix [27]. There are also reports that show the efficacy of CTAB over SDS in controlling bacterial biofilms [27]. The present study showed that *Y. lipolytica* biofilms could be removed effectively by SDS compared to CTAB.

**Figure 4:**
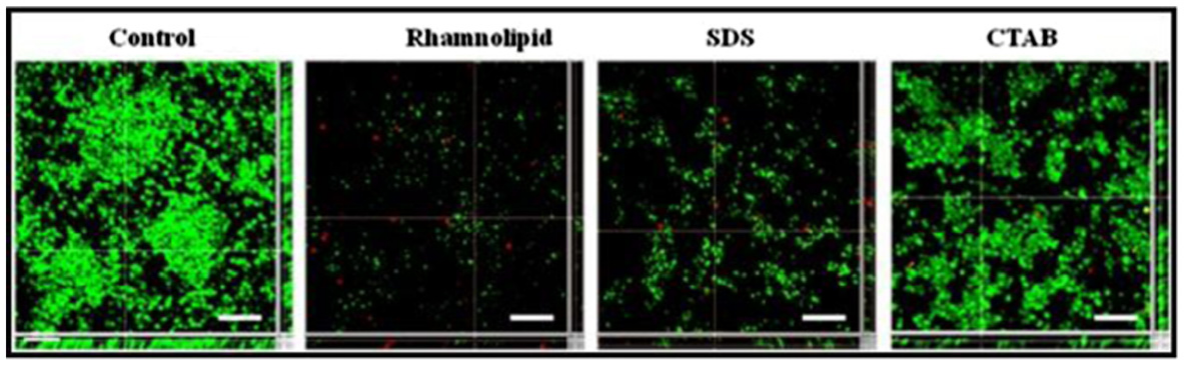
**Representative CLSM images of *Y. lipolytica* NCIM 3589 biofilms.** Observations of control cells and cells treated with sub-MIC concentration of rhamnolipid (2.5% w/v), SDS (0.3% w/v) and CTAB (2.5% w/v) for 1 h, showed live cells stained with SYTO9 and dead cells stained with PI. Bar indicates 30 μm.

Biofilms of *Y. lipolytica* formed on glass slides for 3 days were disrupted by using sub-MIC concentrations of the surfactants. The concentrations of surfactants were selected on the basis of the microtiter plate results. Rhamnolipids effectively disrupted *Y. lipolytica* biofilms on glass surfaces upto 76% with SDS, 53% and with CTAB 38% disruption was observed. The disruption with rhamnolipids was found to be statistically significant (P<0.05) as compared to SDS and CTAB. Rhamnolipids have earlier shown to be effective against bacterial biofilms [14], however there are limited reports on their effect on fungal biofilms. In the present study rhamnolipids were found to be effective in disrupting biofilms of *Y. lipolytica* compared to SDS and CTAB, suggesting their potential application as biofilm disrupting agents (Figure 4). The effectiveness of rhamnolipids against biofilms even at low concentrations makes them a good candidate for therapeutic applications.

## Conclusions

Rhamnolipids have potential to disrupt *Y. lipolytica* biofilms as compared to chemical surfactants, SDS and CTAB. The results suggest potential of rhamnolipid biosurfactants as anti-adhesive and preformed biofilm disrupting agents and their possible role in the treatment of fungal infections.

## Methods

### Culture and growth conditions

A biofilm forming strain of *Y. lipolytica* NCIM 3589 was used in the experiments [6]. Cells were grown in 50 ml YEPD broth (yeast extract 0.3%, peptone 0.5% and dextrose 1%) in 250 ml Erlenmeyer flasks at 30°C on shaker at 150 rpm for 24 h.

### Chemical agents

Rhamnolipid biosurfactant, a mixture of mono and di-rhamnolipids was used [14]. CTAB and SDS were obtained from HiMedia, India. Stock solutions of the surfactants were prepared in sterile distilled water; filter sterilized through 0.22 μ filters and used further for experimentation.

### Determination of MIC and MFC values

MIC of surfactants against *Y. lipolytica* was determined by broth microdilution assay in sterile 96 well microtiter plates (Tarsons, India) [14]. Briefly, pre-grown (36 h) cells of *Y. lipolytica* were added to the microtiter plate wells containing YEPD medium to achieve the final cell numbers of 1 × 10^7^ cells ml^-1^. Surfactants (rhamnolipid, SDS and CTAB) were added to these wells at varying concentrations (0.005 to 10% w/v). The final volume in the microtiter plate wells was maintained to 200 μl. The plates were incubated at 30°C for 48 h and after the incubation period, growth in presence of the surfactants was estimated as OD_600_ using a microtiter plate reader (Multiskan, Thermo Lab systems). Wells without surfactants and those lacking the cells were used as controls. The MFC was determined by streaking the culture grown in presence of different concentrations of the biosurfactant onto YEPD agar plates. The plates were incubated for 48 h and growth was recorded. Minimum inhibitory concentration (MIC) was determined as the lowest concentration without visible growth and the minimum fungicidal concentration (MFC) as the lowest concentration showing no growth on the agar surface. The experiments were performed in triplicate and the mean values were obtained.

### Fungal cell permeability analysis by methylene blue dye exclusion

In order to determine the effect of surfactants on yeast cell permeability, the method of Hammer *et al.* (2004) was followed with few modifications [28]. *Y. lipolytica* cells were grown in YEPD broth for 24 h. The cells were harvested, washed twice with sterile distilled water and resuspended in PBS to achieve 10^7^ cells ml^-1^. Aliquots were distributed equally in sterile flasks containing rhamnolipids, SDS or CTAB at MIC and sub-MIC concentrations. The flasks were incubated at 30°C under shaking conditions. After 1 h, 100 μl of these samples were withdrawn and to this 20 μl of 0.05% w/v methylene blue (prepared in sterile distilled water) was mixed and incubated for 1 min at room temperature. Methylene blue stained samples were placed onto the glass slides and the cells were examined microscopically by using an Axio Scope-A1 microscope with a photographic attachment (ProgRes ® Capture Pro 2.7) at a magnification of 400×. A minimum of 500 cells in consecutive visual fields were examined and the percentage stained cells was calculated manually [28]. Cells untreated with the surfactants were used as controls for the experiment.

### Effect of surfactant pre-coating on biofilm formation

Surfactants (100 μl containing 0.3-10% w/v concentration) were added to wells of the polystyrene microtiter plates and incubated for 12 h at 4°C to facilitate effective coating [23]. After the incubation period, the wells were emptied of residual surfactants, rinsed with sterile distilled water and air dried in a laminar air flow for 5 min. Cells (100 μl containing ~10^7^ cells ml^-1^) of *Y. lipolytica* were added to the microtiter plate wells and incubated for 24 h at 30°C. After the incubation period, the microtiter plate wells were emptied of the non-adherent cells and the plates were rinsed with sterile distilled water. The adherent cells were quantified by using the crystal violet assay [14]. All experiments were carried out in triplicates with two biological replicates and average values indicating standard deviation are presented here.

### Disruption of preformed biofilms

*Y. lipolytica* biofilms were allowed to form in sterile polystyrene 96 well microtiter plate wells for 3 days [6]. After the incubation period, planktonic cells were removed and varying concentrations (0.3-10% w/v) of rhamnolipid, SDS and CTAB were individually added to the wells. The plates were further incubated at 30°C for 1, 2 or 3 h. After each time interval, the microtiter plate wells were emptied of the non-adherent cells and rinsed with sterile distilled water. The residual biofilms were quantified by using the standardized crystal violet assay [14]. All experiments were performed in triplicates with two biological replicates and the data is presented as average values indicating standard deviation.

### Confocal laser scanning microscopy (CLSM)

*Y. lipolytica* biofilms were formed on sterile microscopic glass slides as described earlier [29]. Cells were inoculated in sterile petriplates containing the growth medium (YEPD) to reach a cell density of 1 × 10^7^ cells ml^-1^. Sterile microscopic glass slides were placed in the petriplates and incubated on a rocker at 30°C for 3 days. After the growth period, glass slides were removed and placed in another petri dish containing growth medium supplemented with 0.3% w/v concentration of rhamnolipids, SDS or CTAB. Biofilms were treated for 1 h in presence of the surfactants. Untreated biofilms were used as controls and the biofilm coverage thus obtained was considered to be 100%. The slides were removed, rinsed twice with sterile distilled water and stained with LIVE/DEAD BacLight staining kit containing SYTO9 and Propidium Iodide (PI) (Molecular Probes, Eugene, Oregon, US) as per the manufacturer’s instructions. The slides were observed under a CLSM (SP2 AOBS, Leica Microsystems, Germany). A 63× 1.2 NA water immersion objective was used with 488 nm Ar laser excitation and 500–640 nm band pass emission setting. Multiple (20) images were scanned and analyzed using the image processing software; *ImageJ* (http://rsb.info.nih.gov/ij). The observations were made in triplicates and representative images are presented here.

### Statistical analysis

The effect of rhamnolipid on biofilms was analyzed statistically by the Students *t*-test and treatments were considered significantly different if P≤0.05.

## Abbreviations

CTAB, Cetyl-trimethyl ammonium bromide; SDS, Sodium-dodecyl-sulphate; MIC, Minimum inhibitory concentration; MFC, Minimum fungicidal concentration; PTFE, Polytetrafluoroethylene; NCIM, National collection of industrial microorganisms; YEPD, Yeast extract peptone dextrose; OD, Optical density; CLSM, Confocal laser scanning microscopy.

## Competing interests

The authors declare that they have no competing interests.

## Authors’ contribution

DHD and SD performed the experimental work and acquired the data. YVN performed the CSLM analysis. VPV and SSZ made contributions to conception and design. ARK analyzed and interpreted the data. DHD, SD, VPV and SSZ have been involved in drafting the manuscript and revising it critically for important intellectual content. All authors have given final approval for the version to be published.

## Authors’ information

DHD is a currently a postdoctoral fellow at McGill University. SD is a post-graduate student at Institute of Bioinformatics and Biotechnology (IBB). YVN and VPV are Scientists at Biofouling and Biofilm Processes Section, Chemistry Group, Bhabha Atomic Research Centre, BARC Facilities, Kalpakkam, Tamil Nadu 603102 India. ARK and SSZ are Associate Professors at IBB, University of Pune, Pune 411007 India.

## References

[B1] Barth G Gaillardin C Physiology and genetics of the dimorphic fungus Yarrowia lipolytica FEMS Microbiol Rev 1997 19 219 237 10.1111/j.1574-6976.1997.tb00299.x 9167256

[B2] Margesin R Schinner F Bioremediation of diesel-oil-contaminated alpine soils at low temperatures Appl Microbiol Biotechnol 1997 47 462 468 10.1007/s002530050957

[B3] Zinjarde SS Pant A Hydrocarbon degraders from a tropical marine environment Mar Pollut Bull 2002 44 118 121 10.1016/S0025-326X(01)00185-0 11980445

[B4] Kurtzman CP Fell JW Yarrowia van de Walt and von Arx. Descriptions of telomorphic ascomycetous genera and species The yeasts: a taxonomic study 1998 4 Elsevier Science, Amsterdam 420 421

[B5] Agarwal S Thakur K Kanga A Singh G Gupta P Catheter-related candidemia caused by Candida lipolytica in a child with tubercular meningitis Indian J Patho Microbiol 2008 51 298 300 10.4103/0377-4929.41709 18603717

[B6] Dusane DH Nancharaiah YV Venugopalan VP Kumar AR Zinjarde SS Biofilm formation by a biotechnologically important tropical marine yeast isolate, Yarrowia lipolytica NCIM 3589 Water Sci Technol 2008 58 1221 1229 10.2166/wst.2008.479 18845860

[B7] Chen X Stewart PS Biofilm removal caused by chemical treatments Wat Res 2000 34 4229 4233 10.1016/S0043-1354(00)00187-1

[B8] McDonnell G Russell AD Antiseptics and disinfectants: activity, action, and resistance Clin Microbiol Rev 1999 12 147 179 9880479 10.1128/cmr.12.1.147 PMC88911

[B9] Vieira DB Carmona-Ribeiro AM Cationic lipids and surfactants as antifungal agents: mode of action J Antimicrobial Chemother 2006 58 760 767 10.1093/jac/dkl312 16885181

[B10] Banat IM Franzetti A Gandolfi I Bestetti G Martinotti MG Fracchia L Smyth TJ Marchant R Microbial biosurfactants production, applications and future potential Appl Microbiol Biotechnol 2010 87 427 444 10.1007/s00253-010-2589-0 20424836

[B11] Rodrigues L Banat IM Teixeira J Oliveira R Biosurfactants: Potential applications in medicine J Antimicrob Chemother 2006 57 609 618 10.1093/jac/dkl024 16469849

[B12] Kosaric N Biosurfactants and their application for soil bioremediation Food Technol Biotechnol 2001 39 295 304

[B13] Rivardo F Turner RJ Allegrone G Ceri H Martinotti MG Anti-adhesion activity of two biosurfactants produced by Bacillus spp. prevents biofilm formation of human bacterial pathogens Appl Microbiol Biotechnol 2009 83 541 553 10.1007/s00253-009-1987-7 19343338

[B14] Dusane DH Nancharaiah YV Venugopalan VP Zinjarde SS Rhamnolipid mediated disruption of marine Bacillus pumilus biofilms Colloids Surf B: Biointerfaces 2010 81 242 248 10.1016/j.colsurfb.2010.07.013 20688490

[B15] Busscher HJ Van Hoogmoed CG Geertsema-Doornbusch GI van der Kuijl-Booij M van der Mei HC Streptococcus thermophilus and its biosurfactants inhibit adhesion by Candida spp. on silicone rubber Appl Environ Microbiol 1997 63 3810 3817 9327543 10.1128/aem.63.10.3810-3817.1997 PMC168689

[B16] Glover ER Smith RR Jones VM Jackson KS Rowlands CC An EPR investigation of surfactant action on bacterial membranes FEMS Microbiol Lett 1999 177 57 62 10.1111/j.1574-6968.1999.tb13713.x 10436922

[B17] Ortiz A Aranda FJ Teruel JA Interaction of dirhamnolipid biosurfactants with phospholipid membranes: a molecular level study Adv Exp Med Biol 2010 672 42 53 10.1007/978-1-4419-5979-9_3 20545272

[B18] Aranda FJ Espuny MJ Marques A Teruel JA Manresa A Oritz A Domain formation by a Rhodococcus sp. biosurfactant trehalose lipid incorporated into phosphatidylcholine membranes Langmuir 2007 23 2700 2705 10.1021/la061464z 17662234

[B19] Kim BS Lee JY Hwang BK In vivo control and in vitro antifungal activity of rhamnolipid B, a glycolipid antibiotic, against Phytophthora capsici and Colletotrichum orbiculare Pest Management Sci 2000 56 1029 1035 10.1002/1526-4998(200012)56:12<1029::AID-PS238>3.0.CO;2-Q

[B20] Sotirova AV Spasova DI Galabova DN Karpenko E Shulga A Rhamnolipid–biosurfactant permeabilizing effects on Gram-positive and Gram-negative bacterial strains Curr Microbiol 2008 56 639 644 10.1007/s00284-008-9139-3 18330632

[B21] King AT Davey MR Mellor IR Mulligan BJ Lowe KC Surfactant effects on yeast cells Enzyme Microbial Technol 1991 13 148 153 10.1016/0141-0229(91)90171-6

[B22] Komor E Weber H Tanner W Greatly decreased susceptibility of nonmetabolizing cells towards detergents Proc Natl Acad Sci USA 1979 76 1814 1818 10.1073/pnas.76.4.1814 377284 PMC383482

[B23] Das P Mukherjee M Sen R Antiadhesive action of a marine microbial surfactant Colloids Surf B: Biointerfaces 2009 71 183 186 10.1016/j.colsurfb.2009.02.004 19285837

[B24] Meylheuc T van Oss CJ Bellon-Fontaine MN Adsorption of biosurfactant on solid surfaces and consequences regarding the bioadhesion of Listeria monocytogenes LO28 J Appl Microbiol 2001 91 822 832 10.1046/j.1365-2672.2001.01455.x 11722659

[B25] Nesbitt WE Doyle RJ Taylor KG Hydrophobic interactions and the adherence of Streptococcus sanguis to hydroxyapatite Infect Immun 1982 38 637 644 6292108 10.1128/iai.38.2.637-644.1982 PMC347787

[B26] Schott H Young YC Electrokinetic studies of bacteria II: effect of cetyltrimethylammonium bromide on Streptococcus faecalis J Pharm Sci 1972 61 762 765 10.1002/jps.2600610518 4624709

[B27] Simoes M Simoes LC Machado I Pereira MO Vieira MJ Control of flow-generated biofilms using surfactants-evidence of resistance and recovery Food Bioproducts Process 2006 84 338 345 10.1205/fbp06022

[B28] Hammer KA Carson CF Riley TV Antifungal effects of Melaleuca alternifolia (tea tree) oil and its components on Candida albicans, Candida glabrata and Saccharomyces cerevisiae J Antimicrobial Chemother 2004 53 1081 1085 10.1093/jac/dkh243 15140856

[B29] Dusane DH Rajput JK Kumar AR Nancharaiah YV Venugopalan VP Zinjarde SS Disruption of fungal and bacterial biofilms by lauroyl glucose Lett Appl Microbiol 2008 47 374 379 10.1111/j.1472-765X.2008.02440.x 19146524

